# GREATER DEMENTIA SEVERITY IS ASSOCIATED WITH INCREASED RISK OF POTENTIALLY PREVENTABLE READMISSIONS DURING HOME HEALTH CARE

**DOI:** 10.1093/geroni/igz038.439

**Published:** 2019-11-08

**Authors:** Sara Knox, Brian Downer, Allen Haas, Addie Middleton, Kenneth Ottenbacher

**Affiliations:** 1 MGH Institute of Health Professions, Boston, Massachusetts, United States; 2 University of Texas Medical Branch, Galveston, TX, Galveston, Texas, United States; 3 University of Texas Medical Branch, Galveston, Texas, United States; 4 Medical College of South Carolina, Charleston, South Carolina, United States

## Abstract

Approximately 14.0% of Medicare beneficiaries are readmitted to a hospital within 30-days of home health admission. Individuals with dementia account for 30% of all home health care admissions and are at high-risk for rehospitalizations. Our primary objective was to determine the association between dementia severity at admission to home health and 30-day potentially preventable readmissions (PPR) during home health care. A secondary objective was to develop a dementia severity category from OASIS items based on the Functional Assessment Staging Tool (FAST). Retrospective cohort study of 124,119 Medicare beneficiaries receiving home health (7/2013 – 6/2015) and diagnosed with dementia (ICD-9 codes). The primary outcome was 30-day PPR during home health. The predictor variable of dementia severity was categorized into six levels (non-affected to severe). The overall rate of 30-day PPR was 7.6% (95% CI 7.4, 7.7) but varied by patient and health care utilization characteristics. After adjusting for sociodemographic and clinical characteristics, patients classified as stage 6 and stage 7 had 1.36 (95% CI 1.28, 1.45) and 1.90 (95% CI 1.59, 2.26) times higher odds to experience a 30-day PPR compared to patients classified as stage 1-2. Dementia severity in the later stages is associated with increased risk for PPR. Development of a dementia severity category based on OASIS items and the FAST is feasible. Future research is needed to determine effective strategies for decreasing PPR during home health for individuals with severe dementia. Future research is needed to validate the proposed dementia severity categories used in this study.

